# Chimeric antigen receptor (CAR) modified T Cells in acute myeloid leukemia: limitations and expectations

**DOI:** 10.3389/fcell.2024.1376554

**Published:** 2024-04-17

**Authors:** Beatriz Guijarro-Albaladejo, Cristina Marrero-Cepeda, Eduardo Rodríguez-Arbolí, Belén Sierro-Martínez, José Antonio Pérez-Simón, Estefanía García-Guerrero

**Affiliations:** ^1^ Instituto de Biomedicina de Sevilla, IBiS/Hospital Universitario Virgen del Rocío/CSIC/Universidad de Sevilla, Servicio de Hematología, Hospital Universitario Virgen del Rocío, Seville, Spain; ^2^ Unidad de Gestión Clínica de Hematología, Instituto de Biomedicina de Sevilla, IBiS/Hospital Universitario Virgen del Rocío/CSIC/Universidad de Sevilla, Seville, Spain

**Keywords:** CAR-T cell, acute myeloid leukemia, immunotherapy, clinical trials, target antigens

## Abstract

Acute myeloid leukemia (AML) is an aggressive hematologic malignancy with a poor prognosis despite the advent of novel therapies. Consequently, a major need exists for new therapeutic options, particularly for patients with relapsed/refractory (R/R) AML. In recent years, it has been possible to individualize the treatment of a subgroup of patients, particularly with the emergence of multiple targeted therapies. Nonetheless, a considerable number of patients remain without therapeutic options, and overall prognosis remains poor because of a high rate of disease relapse. In this sense, cellular therapies, especially chimeric antigen receptor (CAR)-T cell therapy, have dramatically shifted the therapeutic options for other hematologic malignancies, such as diffuse large B cell lymphoma and acute lymphoblastic leukemia. In contrast, effectively treating AML with CAR-based immunotherapy poses major biological and clinical challenges, most of them derived from the unmet need to identify target antigens with expression restricted to the AML blast without compromising the viability of the normal hematopoietic stem cell counterpart. Although those limitations have hampered CAR-T cell therapy translation to the clinic, there are several clinical trials where target antigens, such as CD123, CLL-1 or CD33 are being used to treat AML patients showing promising results. Moreover, there are continuing efforts to enhance the specificity and efficacy of CAR-T cell therapy in AML. These endeavors encompass the exploration of novel avenues, including the development of dual CAR-T cells and next-generation CAR-T cells, as well as the utilization of gene editing tools to mitigate off-tumor toxicities. In this review, we will summarize the ongoing clinical studies and the early clinical results reported with CAR-T cells in AML, as well as highlight CAR-T cell limitations and the most recent approaches to overcome these barriers. We will also discuss how and when CAR-T cells should be used in the context of AML.

## 1 Introduction

Acute myeloid leukemia (AML) is characterized by the uncontrolled proliferation of myeloid progenitors, leading to the accumulation of blasts into bone marrow and blood. It accounts for 15%–20% of acute leukemias in the pediatric population, and stands as the predominant form of acute leukemia in adults (Lagunas-Rangel et al., 2017). AML originates from a hematopoietic progenitor cell that undergoes various mutations and genomic rearrangements, endowing the myeloid blasts with proliferative and survival advantages, alter apoptosis and cell differentiation and promote epigenetic modifications (DiNardo and Cortes, 2016). Current intensive treatment for AML includes cytotoxic chemotherapy, and autologous or allogeneic stem cell transplantation, and since the approval of new targeted therapies such as FLT3 inhibitors, IDH inhibitors, BCL2 inhibitors or anti-CD33 antibody drug conjugate, personalized treatment strategies have become possible for a subgroup of patients (Döhner et al., 2022). However, the standard of care for AML, involving intensive chemotherapy with anthracyclines and cytarabine, has remained unchanged for several decades (Network, 2019). Additionally, only 50%–70% of patients achieve complete remission (CR) (Network, 2019), and many patients experience relapse after treatment, resulting in an overall survival rate of approximately 30% at 3–5 years (Halpern et al., 2023). Therefore, there is a need to develop more efficient therapies for AML, and different strategies are being tested in preclinical and clinical studies (Kantarjian et al., 2021).

In 1987, Dr. Yoshikazu Kurosawa and his team described the concept of chimeric T cell receptors (Kuwana et al., 1987). A few years later, first-generation Chimeric Antigen Receptors (CAR) T cell therapy was developed, and the first clinical trials were performed in the context of solid tumors, with patients with ovarian, renal cancer and neuroblastoma. CARs are synthetic receptors comprising an antigen-binding domain, derived from a single-chain variable fragment (scFv) of a monoclonal antibody. This domain is linked to an intracellular signaling domain formed by a T cell receptor (TCR)-derived CD3z chain. In this sense, CAR-T cell therapy facilitates the engineering of lymphocytes to eliminate and recognize cells in a human leukocyte antigen (HLA)-independent manner. However, the effectiveness of first-generation CAR-T cells was disappointing, and led to next-generation CAR-T cells (Charrot and Hallam, 2019). Second and third-generation CAR-T cells further integrate costimulatory domains such as 4-1BB or CD28 into their structure to increase the proliferation and cytokine production (Sterner and Sterner, 2021). In 2017, the FDA approved the first CAR-T cell therapy (Tisagenlecleucel) for pediatric and young adult acute lymphoblastic leukemia (ALL). Since then, other CAR-T cell products have been approved for different B cell malignancies and multiple myeloma (Mitra et al., 2023). Substantial efforts have been dedicated to the development of an effective CAR-T therapy for the treatment of AML, inspired by the positive results witnessed in B-cell malignancies (Bachy et al., 2022; Costa et al., 2022; Melenhorst et al., 2022). Nevertheless, the application of CAR-T cell therapy into the AML context has not proven to be as successful as in other hematological malignancies (Marvin-Peek et al., 2022) and to date, no CAR-T cell product has been approved by regulatory agencies for the treatment of AML. This is primarily due to the challenge of identifying a target antigen with expression confined specifically to AML blasts (Schorr and Perna, 2022) and because of the immunosuppressive microenvironment of the disease (Maucher et al., 2021).

In this review, we will focus on clinical trials and outcomes reported so far regarding CAR-T cell treatment in AML, and we will also analyze limitations and new approaches to overcome them with next-generation CAR-T cells. Finally, we will discuss about the role that CAR-T cell therapy plays in the landscape of AML treatment, and assess its potential as an effective conditioning method.

## 2 CAR-T cells in clinical trials for AML

The goal of CAR-T cell therapy is to selectively eliminate tumor cells while sparing non-tumor tissue, and to provide sustained anti-tumor immunosurveillance that prolongs remission. Therefore, selection of a suitable antigen is essential for the development of an optimal CAR-T cell product. As the recognition of an antigen by the CAR is HLA-independent, the target must be expressed in the cell surface. Besides, the antigen must be homogeneously expressed in tumor cells and have an essential role in their proliferation and survival to avoid escape from CAR-T cells recognition. The ideal target for an AML-directed CAR should be highly expressed on AML blasts and leukemic stem cells (LSCs), but not expressed on healthy tissues or normal hematopoietic stem cells (HSCs). Such a target would maximize the immune-mediated anti-leukemic effect while minimizing the potential for off-target side effects. Currently, several CAR-T targets are under investigation in AML, and ongoing CAR therapy trials in AML have been described.

### 2.1 CD123

The alpha chain of the human interleukin-3 receptor (IL-3Rα), also known as CD123, is frequently overexpressed in several hematological malignancies, including AML where it is expressed on blasts from patients (77,9%) and LSCs (80,7%) (Ehninger et al., 2014). However, CD123 is also expressed on normal HSCs, although its expression varies regarding on the source from which HSCs derive, whether they came from cord blood or bone marrow (Testa et al., 2014). Because of the overexpression on AML blasts and LSCs, CD123 has been considered as a target in AML, and agents that specifically interact with this receptor have been developed, such as bispecific antibodies, antibody-drug conjugates or naked antibodies (Pelosi et al., 2023). In this sense, CD123 has been considered as a suitable antigen for CAR-T therapy in AML, and several clinical trials are being developed targeting this antigen.

Yao et al. (2019) presented a 25-year-old patient with FUS-ERG + AML who relapsed after allogeneic stem cell transplantation (alloHSCT) and received donor-derived CD123 CAR-T cells as part of the conditioning regimen for haploidentical HSCT. They used a reduced-intensity conditioning regimen based on therarubicin, teniposide, fludarabine and busulfan (TVFB) plus CD123 CAR-T cells 1 day after preconditioning. The patient received a single dose of 1 × 10^6^ CAR-T cells/kg, and 4 days after CAR-T infusion he received prophylaxis anti-thymocyte globulin for graft versus host disease (GvHD). The stem cells and the CAR-T cells were obtained from the father of the patient. The patient achieved CR with incomplete hematologic recovery (CRi), full donor chimerism and myeloid engraftment. As side effects of these procedures, the patient had a grade (G) 4 cytokine release syndrome (CRS), G 4 acute GvHD and infections and died on day 56.

Ehninger et al. (2022) performed a phase Ia trial in relapsed/refractory (R/R) CD123+ AML patients using next-generation CAR-T cells with CD28 costimulatory domain and a CD123 targeting module (TM). Both the CAR-T and the TM were individually inert and their activity was only achieved when both of them were present. They published results in 14 patients with median age 65 (range 18–80) and a median number of 3 prior treatment lines (range 1–7), and 4 of them had a prior alloHSCT. They used standard fludarabine/cyclophosphamide (Flu/Cy) lymphodepletion on day −5 and −3. Two patients withdrew from the trial due to disease progression or dose limiting toxicities (DLT). Twelve patients completed the treatment, 12 patients had CRS (most of them G 1–2, only one with G 3) and 1 patient presented grade 2 immune effector cell-associated neurotoxicity syndrome (ICANS), but treatment toxicities were resolved after TM deprivation. Regarding outcomes, most patients had hematological recovery and 10 patients reduced bone marrow blasts count. They observed 2 CRi and 4 partial remissions (PR), and one patient with prior CR measurable residual disease (MRD) positive converted to MRD negative.

Another phase I trial by Budde et al. (2020) analyzed the activity of CD123 CAR-T cell therapy, generated from autologous or allogenic T lymphocytes. They treated 7 patients, with a median of 4 (range 4–10) prior lines of treatment and all of them with at least one prior alloHSCT. All patients received lymphodepletion (mostly Flu/Cy) and they were divided into two cohorts: 2 patients in dose level (DL) 0 (50 × 10^6^ CAR + T) and 5 patients in DL 1 (200 × 10^6^ CAR + T). All patients achieved T-cell expansion peak within the first 14 days. They did not develop remarkable toxicities (no G 3 or above CRS or ICANS, no DLTs, no treatment-related cytopenias longer than 12 weeks). Two patients had morphologic leukemia-free state (MLFS): one of them (who received DL 0) persisted 70 days and 3 months later the patient received a second infusion, the other one (with DL 1) improved to CR at day 84. Another patient in DL 1 had CRi at day 28. The remaining three patients in DL 1 had stable disease (SD).

Naik et al. (2022) reported a phase I study in pediatric patients with R/R AML receiving CD123 autologous CAR-T cells with a CD28.z signaling domain and a CD20 safety switch. They included 12 patients with median age 17 (range 12–21 years) of whom 11 had received a prior alloHSCT and treated 5 of them (2 with DL 1 and 3 with DL 2). There was no G 2 or above CRS or ICANS. The two patients treated with DL 1 did not respond to treatment. Two patients on DL 2 responded to treatment: one patient with isolated extramedullary disease achieved CR 4 weeks after treatment, although then subsequently relapsed due to the loss of the CAR-T cell; in other patient they observed reduction in blasts count without achievement of CR. One patient was infused off protocol with DL 2 and achieved CR at day 28 with low level MRD.

A phase I trial by Sallman et al. (2022a) enrolled 16 patients, with a median of 4 prior lines of treatment (range 3–9) or alloHSCT (9 patients), who received lymphodepletion with either Flu/Cy or Flu/Cy with alemtuzumab (A) followed by CD123 CAR-T cell therapy at different DLs. Median age was 57 years (range 18–65). Fifteen patients developed CRS, 3 of them were G 4 or above, and one patient had G 3 ICANS. Four out of the 16 patients showed evidence of CAR-T cell activity. In the Flu/Cy arm, there was one patient with SD and another with MLFS on day 28, while in the Flu/Cy+A arm, one patient had SD with substantial blast count reduction, and one patient had MRD negative CR that persisted 8 months after treatment. The Flu/Cy+A arm had better lymphodepletion and cell expansion than the Flu/Cy arm.

The last study in this section (Shelikhova et al., 2022) included three pediatric patients with prior alloHSCT. Two of them received allogeneic CD33 CAR-T cells and the other allogeneic CD123 CAR-T cells, all of them were previously treated with fludarabine and cytosine arabinoside (FlA) chemotherapy and Flu/Cy for lymphodepletion. All patients developed CRS (G 3 or below) and G 3–4 hematological toxicities, and two had G 1–2 ICANS. The median time to CAR-T cell peak expansion was 14 days. All three patients achieved CR, two of them with MRD negative, although both relapsed at 2 and 4 months. The MRD positive patient underwent a second haploidentical HSCT and had MRD negative disease at day 100.

### 2.2 CD33

CD33 is a sialic acid-binding immunoglobulin-related lectin which works as a transmembrane receptor on hematopoietic cells. It works as an inhibitory receptor when phosphorylated, and it inhibits the production of pro-inflammatory cytokines (Molica et al., 2021). CD33 is expressed in around 90% of blasts in AML patients regardless of prognosis (Ehninger et al., 2014). Although HSCs also express CD33, its expression into the AML context seems to be lower than within a normal hematopoietic environment (Taussig et al., 2005). Targeted therapy in AML against CD33 has been pursued for years, and validation of this approach comes from the monoclonal antibody gemtuzumab ozogamicin (Molica et al., 2021). Besides, several clinical trials targeting CD33 with CAR-T cell therapy are being conducted.

Sallman et al. (2022b) conducted a phase I/Ib trial using an autologous CD33 CAR-T cell therapy. They treated 20 AML patients and 4 additional patients with other hematologic malignancies. Patients were divided into two groups: those who did not receive lymphodepletion (C1, 10 patients) and those who did (C2, 14 patients). The median of prior lines of treatment was 3 (range 1–9) and 15 patients had undergone prior alloHSCT. Seventeen patients developed CRS: 10 of them were G 1 (3 in C1, 7 in C2), 6 were G 2 (3 in C1 and 3 in C2) and only 1 patient in C1 presented G 3 CRS. Peak expansion was higher in patients with lymphodepletion than in those without. In patients from both cohorts CAR-T cells persisted in blood for up to 7 months after infusion. In cohort 2, one patient reached CRi and was bridged to alloHSCT, and he achieved MRD negative CR which persisted at 18 months post alloHSCT; another patient accomplished CR with partial hematologic recovery (CRh) and one patient with isolated extramedullary disease achieved PR. In cohort 1, one patient achieved durable SD lasting more than 7 months after infusion with persistence of the CAR-T.

Wang et al. (2015) treated one AML patient with autologous CD33 CAR-T cells. The patient was administered a total of 1.12 × 10^9^ CAR T cells in four different infusions. They did not use any conditioning chemotherapy. The patient developed G 1–2 CRS and persistent pancytopenia. The bone marrow blast count decreased (PR), but then the disease progressed, and the patient died 13 weeks after the infusion.

Another study performed by Tambaro et al. (2021) described the treatment of R/R AML patients with autologous CAR-T cells modified to express a CD33-targeted CAR with 4-1BB and CD3ζ endo-domains and co-expressed with truncated human epidermal growth factor receptor (HER1t). Ten patients were enrolled with median age 30 (range 18–73), three had received a prior alloHSCT, and median number of prior lines of treatment was 5 with a range from 3 to 8, but only 3 patients were infused. This was due to failures on the manufacturing process or due to disease progression. One patient had G 2 CRS and another one developed G 3 CRS and G 2 ICANS. They reported other toxicities, related and unrelated to the CAR-T, such as tumor lysis syndrome, infections or analytic alterations. However, they did not observe any anti-leukemic activity and the three patients experienced disease progression and died.

Huang et al. (2022) published a clinical trial with CD33 CAR-NK therapy applied after preconditioning with Flu (30 mg/m2) and Cy for 3–5 days. They included 10 R/R AML patients between 18 and 65 years old (median age 44.5) with a median of prior treatment regimens of 5 (range 3–8). They used lyphodepletion regimen with Flu/Cy and patients were treated with three different DL: 6 × 10^8^, 1.2 × 10^9^ and 1.8 × 10^9^ cells per round. G 1 CRS was described in 7 patients and only one patient had G 2 CRS. Six patients reached MRD negative CR at day 28. One patient underwent alloHSCT after CAR-NK. However, only two patients maintained MRD-CR until last follow up, three of them died and five patients relapsed from disease.

### 2.3 CLL-1 (CLEC12A)

C-type lectin-like receptor 1 (CLL-1) belongs to the family of C-type lectin-like receptors, and it plays a pivotal role during inflammation, as it is a regulator of granulocyte and monocyte function (Ma et al., 2019). CLL-1 is proposed as a CAR-T cell therapy target in AML because its expression on primary AML lines varies from 77.5%–90% and also in LSCs, whereas HSCs barely express CLL-1 (2.5%) (Ma et al., 2019).

Liu et al. enrolled children and adults in a phase I clinical trial testing a dual CLL-1/CD33 CAR-T cell therapy (Liu et al., 2018). At the European Hematology Association Meeting 2020, they reported data from 9 patients with R/R AML who received CAR-T cells manufactured from autologous T cells or from HLA matched sibling donors. The median age was 32 years old (range 6–48). All patients received Flu/Cy lymphodepletion and they were treated with dose escalation 1–3x10^6^ with a single or split dose: 4 patients received DL 1 1 × 10^6^/kg, 3 patients received DL 2 2 × 10^6^/kg and 2 received DL 3 3 × 10^6^. Regarding toxicities, 8 patients developed CRS: 3 G 1, 3 G 2 and 2 G 3 and 4 patients developed ICANS. After 4 weeks, 7 patients achieved remission with MRD negativity (Liu et al., 2020), and one 6-year-old patient who received Flu/Cy lymphodepletion achieved MRD negative CR on day 19 and underwent an alloHSCT.

Pei et al. (2023) reported data from a prospective study in which they included seven pediatric patients with R/R AML and the median age of patients was 8.4 years (5.8–13.5 years). From these patients, four were treated with CD28/CD27 anti-CLL-1 CAR-T cells and three were treated with 4-1BB anti-CLL-1 CAR-T cells, and all received Flu/Cy lymphodepletion chemotherapy. Patients received different doses from 0.94 to 1.98 × 10^6^ cells per kilogram. Three of the four patients into the CD28/CD27 group achieved CR within the first 3 months with MRD negativity (overall response rate of 75%), while two from three patients (one of them with MRD negativity) achieved CR into the 4-1BB group (overall response rate of 67%). Regarding toxicities, all patients experienced G 1 or 2 CRS, and one patient developed G 2 ICANS. One CR patient received an alloHSCT and died 7 months later due to GvHD. Three other patients died of disease progression or relapse.

Another study by Jin et al. (2022) reported the efficacy and safety of a CLL-1-targeting CAR-T cell therapy in 10 adult patients with R/R AML. CAR-T cell source and construct design were not specified. The median number of prior lines of therapy was 5 (range 2–10), and 5 patients had undergone alloHSCT. Flu/Cy lymphodepleting conditioning chemotherapy was employed. Different DL were used ranging from 1 × 10^6^ - 2 × 10^6^ cells/kg. Peak expansion was achieved within 2 weeks. All patients developed CRS, with 6 cases considered high-grade events, although every case was controlled with either tocilizumab or corticosteroids. No patient developed CAR-T cell-related neurotoxicity. Notably, all patients developed severe pancytopenia, and 2 patients died of infections in the setting of unresolved agranulocytosis. CR/CRi rate was 70%. Six patients underwent alloHSCT at a median of 20 days after infusion (range 18–34), and 1 patient remained in CR beyond 6 months without transplant consolidation.

Ma et al. treated two patients who relapsed after multiple treatment lines, including alloHSCT and CD38 CAR-T with PD-1 silenced anti-CLL-1 CAR-T therapy (Ma et al., 2022). The first patient was a 28-year-old male who received lymphodepletion with Cy and afterwards he was treated with 1 × 10^7^/kg of CLL-1 CAR-T cells by dose escalation during 3 days. He developed CRS G 1 and no signs of ICANS were observed. He received an alloHSCT after the CAR-T and he maintained CR for 8 months. The second patient was also a 28-year-old male and he received cytoreduction chemotherapy with decitabine, cytarabine, cladribine and granulocyte colony-stimulating factor (D-CLAG). He was treated with 5 × 10^6^ cells/kg for 2 days. Regarding toxicities, the patient developed G 2 CRS without symptoms of ICANS. He achieved morphological MRD negative CRi on day 28.

### 2.4 NKG2D ligands

NKG2D is an activating receptor expressed on T and NK cells which recognizes a group of ligands (NKG2D-L) which are upregulated in malignant transformation, including AML, and they are barely expressed on healthy tissues (Hilpert et al., 2012). Therefore, the development of anti-NKG2D-L CAR-T cells by incorporating the NKG2D sequence into the CAR has been proposed as a therapeutic option for AML.

Baumeister et al. (2019) enrolled 12 patients with median age of 70 (44–79 years), 7 of them with AML, in a phase I study of autologous NKG2D CAR-T cells. The median of prior lines of therapy was 1 (range 1–4). They did not use lymphodepletion and all patients received autologous CAR-T cells which were successfully manufactured on four different DL ranging from 1 × 10^6^ to 3 × 10^7^ viable T cells. There were no CRS nor ICANS, however, no objective responses to the therapy were reported, and median overall survival was 4.7 months.

Sallman et al. (2023) developed an autologous NKGD2 CAR-T cell and they enrolled 25 patients on a phase I study to evaluate this therapy against different hematologic malignancies. Participants were 18 years or older, and they had R/R disease after previous treatments. They evaluated three DLs: 3 × 10^8^, 1 × 10^9^, and 3 × 10^9^ total cells, and they treated 16 patients, from which 12 were AML patients. Regarding toxicities, they observed CRS in 15 patients (94%), five of them were G 3–4 CRS. One patient with AML presented DLT of CRS and finally died. For the rest, the objective response rate at 3 months was 25%. Among responders, two patients with AML received alloHSCT after CAR-T cell therapy and they maintained remission for 5 and 61 months.

### 2.5 CD7

CD7 is a transmembrane glycoprotein which works as a costimulatory receptor for T and NK cells during their development. CD7 is also expressed in blasts and LSCs in around 30% of AML patients (Gomes-Silva et al., 2019), so it has been proposed as a possible target to develop CAR-T cell therapy. However, the development of anti-CD7 CAR-T cells may lead to therapeutical failure due to fratricide effect, and the depletion of CD7 before manufacture is needed, as it has been described that CD7 is not essential for correct T cell development and function (Png et al., 2017).

Hu et al. (2022) developed healthy donor-derived CD7 CAR T cells, in which they knocked out the expression of CD7, HLA-II and TCR with CRISPR/Cas9. They conducted a clinical trial with 12 leukemia/lymphoma patients, one with CD7-positive AML. The patient with AML received lymphodepletion chemotherapy with Flu/Cy and etoposide prior infusion with 2 × 10^6^ cells/kg of CAR-T cells. The patient developed G 2 CRS and G 1 ICANS, and achieved CRi 28 days post infusion.

Rather than taking advantage of gene editing tools to deplete CD7 from CAR-T cells and thus avoiding off target effects, Freiwan et al. were able to develop anti-CD7 CAR-T cells from a naturally occurring population of CD7 negative T cells, which presented high antitumor activity and avoid fratricide effect (Freiwan et al., 2022). This strategy has achieved clinical practice on a phase I clinical trial by Zhang et al. (2023) were they evaluated the efficacy of naturally CD7 negative anti-CD7 CAR-T cells to treat CD7 positive AML patients. They enrolled 10 patients with R/R AML with a median age of 34 (7–63) and median bone marrow blast percentage of 17% (2%–72.2%). After 4 weeks, 7 patients achieved CR and 3 patients showed no response due to the loss of CD7. Among the 7 patients with CR, 3 of them underwent alloHSCT after CAR-T infusion and two of them remained in leukemia-free state on days 752 and 315. Regarding toxicities, 8 patients developed G 1–2 CRS and 2 developed G 3 CRS, and none of the patients have ICANS. However, mostly all patients relapsed due to CD7 loss.

### 2.6 LeY

The antigen Lewis-Y (LeY) is a tumor-associated antigen of the blood-group molecule family. It is not expressed on erythrocytes and it is lowly expressed on healthy tissues (Westwood et al., 2009). However, it is overexpressed in a wide range of tumors, including AML (Goswami and Hourigan, 2017), making it a suitable target for CAR-T cell therapy in AML context.

Ritchie et al. (2013) reported the feasibility and safety of autologous anti-LeY CAR-T cell therapy. They treated four patients, using preconditioning with Flu and patients received 1.3 × 10^9^ total T cells with 14%–38% of positive CAR-T cells. There were no G 3–4 toxicities. Two patients experienced cytogenetic remission, one relapsed and the other enrolled in another trial; one patient had blast count reduction but then the disease progressed.

## 3 Limitations of CAR-T cell therapy in AML

Several patients achieved CR following CAR-T cell therapy targeting CD123, CD33 and CLL-1, and one patient responded targeting CD7. Notably, clinical trials involving anti-NKG2D-L and anti-LeY CAR-T cells reported less favorable outcomes. Despite these initial findings from CAR-T clinical trials in AML, as outlined in Table 1 (CAR-T trials) and Table 2 (CAR-NK trials), these results have proven to be unsatisfactory, particularly when compared with the remarkable outcomes noted in other hematologic malignancies, particularly B-cell malignancies (Bachy et al., 2022; Costa et al., 2022; Melenhorst et al., 2022). Although early clinical results exhibit significant heterogeneity in terms of response rates, these tend to be transient and most patients eventually experience disease progression or relapse. Besides, the follow-up time in most published studies lacks information about long-term responses. One of the main limitations hindering the application CAR-T cell therapy in AML context is the lack of specific antigens with restricted expression on myeloid LSCs to mitigate “on-target off-tumor” toxicity, prolonged myelosuppression, or disease progression (Marvin-Peek et al., 2022). In B-cell malignancies, the B cell aplasia resulting from CD19 CAR-T cell therapy has proven to be clinically manageable (Wat and Barmettler, 2022), with many patients experiencing resolution of the aplasia during the follow-up period (Molinos-Quintana et al., 2023). However, in AML, prolonged myeloablation resulting from the fact that healthy cells also express CAR-T cell target antigens may lead to death due to neutropenic infections and bleeding complications (Mardiana and Gill, 2020). Moreover, the expression of antigens in AML blasts is very variable, and this heterogeneity is one of the main limitations of CAR-T therapy in the AML context (Ediriwickrema et al., 2023), as it is a challenge to selectively target the AML blast without depleting normal HSCs. Therefore, efforts are underway to identify new antigens suitable for CAR-T cell therapy in AML (Ehninger et al., 2014; Perna et al., 2017).

**TABLE 1:** CAR-T clinical trials for the treatment of AML.

Target	Identifier	CAR-T type	Patients	Lymphodepletion	Dosage	Responses	Toxicity
CD123 (Yao et al., 2019)	NA	Donor-derived CART123 as part of conditioning regimen for haplo-HSCT	1	NA	1 dose of 1 × 10^6^ cells/kg	CRi	G 4 CRS
CD123 (Ehninger et al., 2022)	NCT04230265	UniCAR-T with CD28 costimulatory domain and a CD123 TM.	14	Flu/Cy on days −5 and −3	4 different doses with DLT at 500 M CAR-T cells	2 CRi, 1 MRD (+) - > MRD (-), 4 PR.	12 with CRS (1 G 3, 11 G 1–2), 1 with G 2 ICANS
CD123 (Budde et al., 2020)	NCT02159495	Autologous or allogenic CD123CAR-CD28-CD3ζ-EGFRt-expressing T Lymphocytes	7 (18 enrolled)	Mostly Flu/Cy	2 patients DL 0 (50 M CAR+) and 5 DL 1 (200 M CAR+)	2 MLFS (1 that improved to CR), 1 CRi	G 1–2 CRS
CD123 (Naik et al., 2022)	NCT04318678	CD123-CAR with CD28.z signaling domain and a CD20 safety switch	12 (5 with results)	Flu/Cy	1 single dose. 4 DL: DL 1: 3 × 10^5^/kg, DL 2: 1 × 10^6^/kg, DL 3: 3 × 10^6^/kg, DL 4: 1 × 107/kg	1 CR (then relapsed); 1 CR off protocol	No G ≥2 CRS, no ICANS
CD123 (Sallman et al., 2022a)	NCT04106076	Lentiviral vector to express the anti-CD123 and modified to disrupt the TRAC and CD52 genes	16	Flu/Cy or Flu/Cy + alemtuzumab	DL 1: 2.5 × 10^5^/kg, DL2: 6.25 × 10^5^/kg, DL 2i: 1.5 × 10^6^/kg, DL 3: 3.03 × 10^6^/kg	2 SD, 1 MLFS, 1 CR (MRD-)	15 with CRS, 1 with G 3 ICANS
CD 123/CD33 (Shelikhova et al., 2022)	NA	One CD123 allo-CAR-T cells, 2 CD33 CAR-T cells. Both 2-nd generation 4-1BBz CARs	3 children	Flu/Cy	2 patients with 1 × 10^6^ cells/kg and 1 with 5 × 10^5^ cells/kg	3 CR (2 with MRD -). 2 of them relapsed at 2 and 4 months	3 with G ≤3 CRS, 2 with G 1–2 ICANS.
CD33 (Sallman et al., 2022b)	NCT03927261	PRGN-3006 (autologous ultraCAR-T)	24 (20 with AML); 10 without LD (C1), 14 with LD (C2)	Cohort 1 without LD, cohort 2 Flu 30 mg/m2 and Cy 500 mg/m2 days −5 and −3	C1: 1.8 to 50 × 10^6^	C2: 1 CRi bridged to allo (CR MRD- post alloHSCT), 1 CRh and 1 PRC1: 1 durable SD	10 with G 1 CRS (C1 3; C2 7), 6 with G 2 CRS (C1 3; CD 3), 1 with G 3 CRS (C1)
					C2: 4.4 to 83 × 10^6^		
CD33 (Wang et al., 2015)	NCT01864902	Autologous CART-33 cells	1	No LD	1.12 × 10^9^ T cells in 4 doses (38% CAR +)	PR	G 1–2 CRS
CD33 (Tambaro et al., 2021)	NCT03126864	Autologous T cells, modified to express a CD33-targeted CAR with 4-1BB and CD3ζ endo-domains and co-expressed with HER1t	3	NA	0.3 × 10^6^ CD33-CAR-T/kg	No responses	1 with G 2 CRS; 1 with G 3 CRS and G 2 ICANS
CLL-1/CD33 (Liu et al., 2020)	NCT03795779	anti-CLL-1 CAR linked to an anti- CD33 CAR via a self-cleaving P2A peptide	9 patients	Flu/Cy	4 patients DL 1 1 × 10^6^/kg, 3 DL 2 2 × 10^6^/kg and 2 DL3 3 × 10^6^	7/9 were MRD-, 2 with no response	8 with G 1–3 CRS
CLL-1 (Pei et al., 2023)	NCT03222674	Anti-CLL-1 CAR-T cells with co-stimulatory domain (CD28/CD27 versus 4-1BB)	7 children: 4 (CD28/CD27) and 3 (4-1BB)	Flu/Cy	0.94–1.98 × 10^6^ cells/kg	5 CR, 3/4 in the CD28/CD27 group and 2/3 in the 41BB group	7 with G 1–3 CRS
	ChiCTR1900027684						1 with G 1–2 ICANS
CLL-1 (Jin et al., 2022)	ChiCTR2000041054	Anti-CLL-1 CAR-T cells	10	Flu/Cy	1 × 10^6^-2x10^6^ cells/kg	7 CR/CRi; 6 underwent HSCT at a median of 20 days post CAR-T	10 with CRS: 4 mild, 6 severe
CLL-1 (Ma et al., 2022)	NCT04884984	PD-1 silenced anti-CLL-1 CAR-T therapy	20 patients in trial, 2 with published results	Patient 1 LD with Cy; patient 2 cytoreduction chemotherapy with D-CLAG	1 × 10^7^/kg for 3 days; patient 25 × 10^6^ cells/kg for 2 days	Molecular CRi recovery at day 28	G 1 and G 2 CRS
NKG2D (Baumeister et al., 2019)	NCT02203825	Autologous NKG2D CAR-T cells	12 (7 with AML)	No LD	1 × 10^6^ to 3 × 10^7^ viable T cells	OS 4.7 months	No CRS or ICANS
NKG2D (Sallman et al., 2023)	NCT03018405	Autologous NKG2D CAR-T cells	16 (12 with AML)	No LD	3 × 10^8^, 1 × 10^9^, and 3 × 10^9^	3 of 12 (25%) objective responses	5 with G 3–4 CRS, one dose-limiting toxicity
CD7 (Hu et al., 2022)	NCT04538599	CD7 CAR-T cells with 4-1BB coestimulatory domain	12 (1 with CD7^+^ AML)	Flu/Cy + etoposide	Patient with: 2 × 10^6^ cells/kg of CAR-T cells	AML patient: CRi at day 28	AML patient: G 1 CRS
CD7 (Zhang et al., 2023)	NCT049388115	CD7- antiCD7 CAR-T cells	10 CD7 + AML patients	Flu 30 mg/m^2^/d and Cy 300 mg/m^2^/d	4 patients with 5×10^5^/kg and 6 with 1×10^6^/kg	7/10 CR at week four	8/10 G 1–2 and 2/10 G 3 CRS. No ICANS
LeY (Ritchie et al., 2013)	NCT01716364	Autologous CAR anti-LeY	5 (4 received the CAR-T)	Flu	1.3 × 10^9^ total T cells (14%–38% CAR+)	3 with evidence of biological responses	No G3-4 toxicity
						All relapsed, died or had disease progression	

LD, lymphodepletion; HSCT, hematopoietic stem cell transplant; CR, complete remission; CRi, CR, with incomplete count recovery; CRS, cytokine release syndrome; TM, targeting molecule; Flu/Cy, Fludarabine/Cyclophosphamide; DLT, dose-limiting toxicities; MRD, measurable residual disease; PR, partial remission; G, grade; ICANS, immune effector cell-associated neurotoxicity syndrome; DL, dose level; MLFS, morphologic leukemia-free state; SD, stable disease; LD, lymphodepletion; CRh, CR, partial with hematologic recovery.

**TABLE 2:** CAR-NK clinical trials for the treatment of AML.

Target	Identifier	CAR-T type	Patients	Lymphodepletion	Dosage	Responses	Toxicity
CD33 (Huang et al., 2022)	NCT05008575	Anti-CD33 CAR-NK cells	10	Flu 30 mg/m^2^ and Cy 300–500 mg/m^2^ days −5 and −3	3 patients DL 1 (3 doses, 6×10^8^, 1.2×10^9^ and 1.8×10^9^ cells); 2 patients DL 2 (one dose 1.8 ×10^9^ cells) and 4 patients DL 3 (3 doses 1.8 ×10^9^)	6 with CR MRD- at day 28	7 with G 1 CRS, 1 with G 2 CRS

CR, complete remission; MRD, measurable residual disease; G, grade; CRS, cytokine release syndrome.

Another challenge hindering the development of efficient CAR-T cell therapy for AML patients arises from obstacle related to the manufacturing of CAR-T cells from these patients. It has been described that the AML microenvironment exhibits characteristics that render it an immunosuppressive milieu (Maucher et al., 2021). Moreover, it has been observed that there is a high accumulation of myeloid-derived suppressor cells and regulatory T cells in the bone marrow and blood of AML patients, both of which contribute to the immunosuppressive microenvironment of AML by directly suppressing T-cell activation (Mardiana and Gill, 2020). Tumor cells also play a role in inducing immunosuppression in the AML context, as AML blasts are implicated in several pathways leading to immune scape. AML cells secrete various factors that impair T-cell function and promote apoptosis, express inhibitory ligands like programmed death receptor 1 (PD-L1) or T cell immunoglobulin and mucin-containing-3 (TIM-3), and downregulate the expression of MHC molecules, impairing their antigen presentation to the immune cells and resulting in immune evasion (Mardiana and Gill, 2020). Furthermore, patients who are candidates to CAR-T cell therapy have typically undergone multiple lines of treatment and intensive chemotherapy in prior interventions. In some instances, this intensive chemotherapy has been reported to induce DNA damage in AML cells resulting in the downregulation of target antigens (Wu et al., 2021). Despite the limitations of CAR-T cell therapy in treating AML, there is a pressing need to develop new strategies to address the aforementioned limitations.

## 4 New approaches for CAR-T cell therapy in AML

**FIGURE 1:**
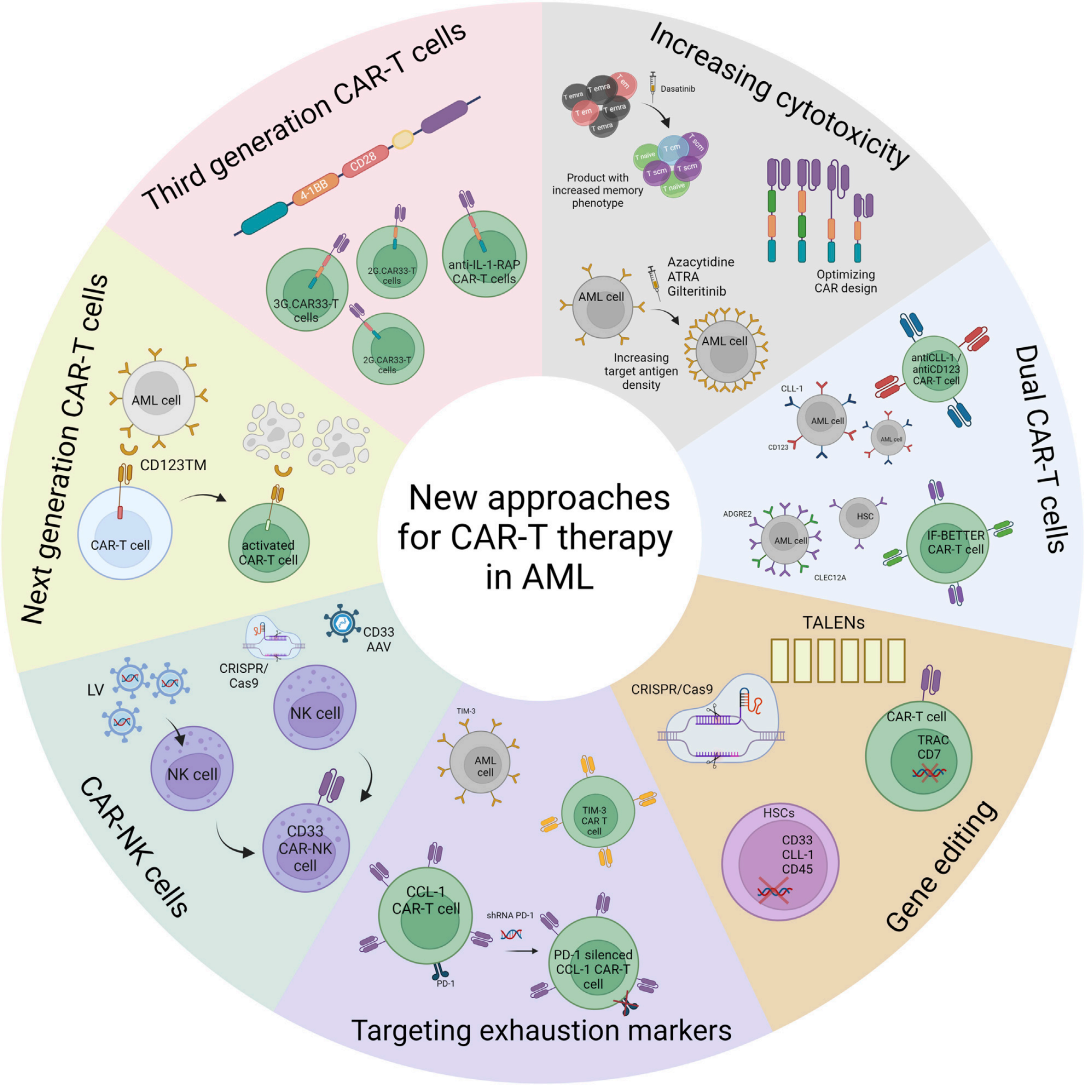
New approaches for CAR-T cell therapy in AML. This figure shows the different approaches that are being developed in preclinical setting to increase the efficacy of CAR-T cell treatment in AML.

In spite of the current limitations in developing effective CAR-T cell therapy for AML, numerous efforts are actively underway to significantly boost CAR-T cell efficacy through various strategies. These new approaches are deriving significant benefits from new advancements in the field, such as enhancing cytotoxicity with combination strategies, developing dual CAR-T cells targeting two antigens to prevent antigen scape and off-tumor toxicities, and creating universal CAR-T cells to mitigate GvHD risks. Additionally, the manufacturing challenges within the immunosuppressive microenvironment of AML are being addressed. In this sense, gene editing tools offer a promising avenue, enabling the generation of allogeneic CAR-T cells from healthy donors. Moreover, the potential to knock out target antigens from HSCs before alloHSCT holds great promise in averting off-tumor toxicities. In this section, we will review new strategies that are being developed with CAR-T cell therapy in AML. These strategies are yielding highly promising results, displaying the remarkable progress in overcoming limitations and enhancing the efficacy of CAR-T cell therapy for AML, and these strategies are summarized on Figure 1.

### 4.1 Increasing CAR-T cell cytotoxicity

One strategy proposed to improve CAR-T potency is to increase the density of the antigen on the AML blast. For this purpose, the research is focused on combination therapy involving CAR-T cells and drugs that enhance the expression of the target antigen on AML cells. In this sense, El Khawanky et al. have described that azacytidine (AZA) treatment upregulates CD123 levels on AML cell lines (el Khawanky et al., 2021). They tested the efficacy of a third-generation CAR against CD123 *in vitro* and in a murine model. They exposed a humanized mice model bearing MOLM-13Luc AML cells to 2.5 mg/kg AZA for three doses, and 24 h after the last dose mice were treated with CD123 CAR-T cells, resulting in a lower tumor burden and longer survival on the AZA-treated group. In this way, other studies have also identified different compounds capable of increasing different AML antigens on leukemic cells and with that, the efficacy of CAR-T cell therapy, as ATRA treatment of AML cell lines and primary samples, which increases CD38 expression on their surface (Yoshida et al., 2016), or the increase of FLT3 in AML cells following treatment with gilteritinib (Li et al., 2022) or crenolanib (Jetani et al., 2018).

The optimization of the CAR design is also crucial for CAR-T therapy success, as it will ensure the correct binding of the CAR-T to their target. In this sense, several strategies to optimize the CAR design have been explored in the AML field. On the one hand, the scFv can be rationally optimized to improve CAR-T cell efficacy targeting specific conformations of the antigen on the target cell surface and, on the other hand, the hinge domain can also be optimized to increase the avidity between the CAR and its ligand. Pérez-Amill et al. showed both strategies. They designed 21 versions of the CAR molecule, in which they changed the order of the heavy and light chains domains in the scFv and the linker length. The versions CD84.02 (VLVH) and CD84.03 (VHVL), which included two different murine scFvs, showed higher efficacy against AML cell lines (Pérez-Amill et al., 2022). In the American Society of Hematology Annual Meeting 2022, Mandal et al. presented a method based on cross-linking mass spectrometry and glycoprotein cell surface capture, which they named “structural surfaceomics,” and they emphasize the importance of determining the structural conformation of the antigen on the target cell. With this technique, they determined that AML cells express an active integrine-β2 conformation, while non-leukemic blood cells expressed the close and resting conformation, making active integrin-β2 a suitable target for CAR-T cell treatment of AML (Mandal et al., 2023). The optimization of the CAR design allows better interaction between the CAR and its ligand, thus increasing the cytotoxic effect of CAR-T cells in the AML context (Leick et al., 2022).

In other hematologic malignancies, it has been proved that CAR-T cell products with increased memory phenotype can achieve better responses than the more differentiated ones (Fraietta et al., 2018; Alvarez-Fernández et al., 2021). Following this concept, Hebbar et al. have developed a strategy to target GRP78 in AML cells with anti-GRP78 CAR-T cells that have been treated with dasatinib during the manufacturing process, thus blocking their differentiation and maintaining a more naïve-like phenotype. With this strategy, a higher antitumor efficacy against AML lines was obtained, and CAR-T cells treated with dasatinib have increased cytotoxicity in repeated antigen stimulation studies (Hebbar et al., 2022).

### 4.2 Dual-CAR-T cells targeting multiple antigens

Downregulation of target antigen in tumor cells has been described as a mechanism of resistance that may lead to relapses in CAR-T treatment (Daver et al., 2021). In this sense dual-CARs appear as a strategy to overcome these limitations, as they are designed to simultaneously target two different antigens.

Ghamari et al. developed a tandem CAR against CD123 and folate receptor β, both upregulated on blasts and LSCs from AML patients (Ehninger et al., 2014; Lynn et al., 2015). This tandem CAR was able to secrete higher levels of interleukins (ILs), specially IFN-γ and IL-2 than single CARs. This increased cytokine secretion leads to increased T-cell activation and a higher percentage of tumor cell lysis, thereby increasing the functionality of tandem CARs compared to single CARs (Ghamari et al., 2021).

Scherer et al. described a ligand.CD70 CAR in combination with CD123 or CLL-1 (Scherer et al., 2022). This CAR includes the CD27 scFv, which is the natural ligand of CD70. This construct in combination with CD123 or CLL-1 showed enhanced antitumor efficacy *in vitro* and in murine models than single CARs, even against low antigen-density tumors.

Although dual CAR-T cells appear to be more potent against AML cells, cells that only express one of the targets are also eliminated, which is a limitation of this strategy since normal cells can also be destroyed. In this sense, one strategy to reduce on-target off-tumor toxicity and overcome antigen scape in heterogeneous AML blasts has been developed by Haubner et al. The IF-BETTER gated CAR-T cells strategy consists of co-targeting two antigens, one with different expression patterns on AML cells and HSCs, and a second antigen with restricted expression on AML cells, thereby reducing on-target off-tumor toxicities (Haubner et al., 2023). The first antigen is ADGRE2, as it showed to be heavily expressed on AML cells (>1.0 × 10^3^ molecules per cell) and lowly expressed on normal HSCs (<1.0 × 10^3^ molecules per cell). The second target is CLEC12A, which they found to be co-expressed with ADGRE2 on LSCs but not on HSCs. They developed a sensitivity-tuned ADGRE2-CAR with a CLEC12A-targeted chimeric costimulatory receptor, which proved greater activity against LSCs without increasing toxicity against HSCs *in vitro* and in murine models, and they have started a phase I clinical trial evaluating this strategy in patients with R/R AML (NCT05748197).

### 4.3 Reducing toxicity of CAR-T cell therapy through gene editing strategies

Regarding CAR-T therapy limitations in AML, the possibility of taking advantage of gene editing tools in order to increase the efficacy of the treatment is a promising approach, as these technologies offer plenty of possibilities to overcome CAR-T limitations (Liu et al., 2019; Dimitri et al., 2022).

One of these approaches consists of deleting CAR-T targets, like CLL-1 or CD33, with CRISPR/Cas9 from HSCs prior to transplant, thus avoiding off-tumor effects from CAR-T therapy and restricting the cytotoxic effect of CAR-T cells just to AML cells (Humbert et al., 2018; Kim et al., 2018; Xavier-Ferrucio et al., 2022). Editing approaches consisting of non-essential antigen targeting present, however, two significant limitations. First, the available pool of truly dispensable targets is likely to be narrow. Second, target functional redundancy could facilitate escape phenomena through marker loss or underexpression, without a deleterious impact on leukemic cell fitness. In this sense, Wellhausen et al. (2023) have described a new approach consisting in editing the pan-hematological epitope CD45 from HSCs and T cells. They were able to introduce a mutation with CRISPR base editing into HSCs on the epitope of CD45 which is targeted by anti-CD45 CAR T cells, which was sufficient to evade recognition while preserving CD45 function. This CD45 edited HSCs could engraft, persist and differentiate in murine models. Secondly, they generated anti-CD45 CAR-T cells from edited T cells, thereby reducing the risk of HSCs killing and fratricide effect. They treated an AML murine model with CD45-edited CAR-T cells achieving tumor control with no presence of AML blasts within 4 weeks of treatment. This approach has also been probed by Casirati et al. (2023), with promising results employing adenine base editors on FLT3, CD123, or KIT.

Nowadays, most of the clinical trials and FDA-approved CAR-T therapies are based on the generation of autologous CAR-T cell products derived from the patients (Rafiq et al., 2020), although this strategy may be limited by the high presence of dysfunctional T cells in some patients (Knaus et al., 2018; Mehta et al., 2021; Calviño et al., 2023). In this sense, generating allogeneic CARs may be a solution to overcome these limitations. The generation of allogeneic products entails the risk of developing GvHD, due to HLA disparities. In this way, Sugita et al. developed an allogenic gene-edited CAR-T cell against CD123, in which they disrupted the TRAC gene with TALENs technology to downregulate the expression of TCRαβ on the T cell surface, leading to minimal toxicities observed against healthy HSCs and normal tissues in murine models (Sugita et al., 2022). This approach has also been employed by Calviño et al. (2023) in the development of allogeneic CAR-T cells targeting CD33. This was achieved by combining CRISPR genome editing techniques and Sleeping Beauty transposons for CAR gene delivery. Consequently, they successfully produced CD33-specific CAR-T cells devoid of HLA-I and TCR genes. This strategy is being evaluated in clinical trials against other hematological malignancies, such as B-ALL (Qasim et al., 2017; Ottaviano et al., 2022).

### 4.4 Targeting exhaustion markers

AML cells upregulate inhibitory markers in order to escape from the immune system, such as PD-L1, which induces an exhausted phenotype on T cells characterized by the expression of lymphocyte-activation gene 3 (LAG3), programmed death receptor 1 (PD-1), cytotoxic T-lymphocyte associated protein 4 (CTLA-4) and TIM3 (Epperly et al., 2020; Zhu et al., 2022). In spite of this, the overexpression of exhaustion markers on AML blasts makes them an interesting target for CAR-T cell therapy, and some groups are developing anti-TIM3 CAR-T cells with high cytotoxicity against AML cell lines and patient-derived AML samples *in vitro* and in murine models (He et al., 2020; Lee et al., 2021).

Recently, we have described a correlation between the presence of PD-1+LAG3+ CD4^+^ CAR-T cells at the peak expansion and event-free survival in patients with ALL and lymphoma treated with Axicabtagene ciloleucel or Tisagenlecleucel (García-Calderón et al., 2023). Other groups have found that the expression of exhaustion markers at the point of leukapheresis and infusion products leads to poor CAR-T efficacy (Fraietta et al., 2018). Besides, another strategy consists in knocking down exhaustion genes in CAR-T cells in order to avoid exhaustion derived from permanent activation of CAR-T cells, which may lead to failure in the treatment of patients with AML. In this way, Lin et al. have designed a third-generation CAR-T targeting CLL-1 and a short hairpin RNA (shRNA) against PD-1, which induced a significant inhibition of PD-1 expression in CAR-T cells (Lin et al., 2021), and this strategy is being deployed in a clinical trial (Ma et al., 2022).

### 4.5 CAR-Natural Killer cells

CAR-NK cells have emerged as an interesting tool for immunotherapy in AML because they exert properties that allow them to kill hematopoietic tumor cells, as they secrete larger amounts of ILs, specially IFN-γ (Khawar and Sun, 2021). One advantage of CAR-NK therapy is that they do not induce GvHD, as they recognize the presence HLA-I molecules into cells surface to spare these cells from cytotoxicity. This feature of NK cells can be used as an anti-tumor strategy, particularly because tumor cells frequently exhibit downregulation or loss of HLA-I molecules (Mehta and Rezvani, 2018). They also induce less CRS and ICANS than CAR-T therapy (Pang et al., 2022). However, their lifespan is low so this therapy will require various doses of treatment to achieve maintained CR (Khawar and Sun, 2021) and CAR-NK manufacturing is more challenging than CAR-T cell expansion. Therefore, there are several strategies under research trying to optimize CAR design and culture protocols to make CAR-NK therapy suitable for clinical practice (Soldierer et al., 2022).

Albinger et al. have developed a CD33-CAR-NK by transduction of peripheral-blood-derived NK cells with lentiviral vectors. They achieved around 30%–60% of transduction rates, and CD33 CAR-NK efficiently eliminated OCI-AML2 and primary AML cells *in vitro*, even at low effector:target ratios, and they were able to kill tumor cells in rechallenge experiments after 3 rounds of repeated antigen stimulation (Albinger et al., 2022). CD33-CAR-NK cells were also evaluated on a humanized murine model injected with OCI-AML2 cells. A high percentage of infiltrating CD33 CAR-NK cells into the bone marrow and spleen of treated mice and a strong reduction of tumor burden at day 21 of treatment was observed, with no signs of CRS or GvHD. Other groups are also trying to develop CAR-NK therapies for the treatment of AML, targeting highly expressed antigens on AML blasts as CD123 (Morgan et al., 2021; Caruso et al., 2022) or CD70 (Choi et al., 2021).

One of the main problems with CAR-NK therapy is the low transduction rates achieved with viral and non-viral methods, because NK cells are very sensitive to foreign DNA delivery, and in consequence CAR expression is low. To overcome this issue, Kararoudi et al. developed a method using CRISPR/Cas9 and adeno-associated vectors (AAVs) for specific integration on NK cells (Kararoudi et al., 2022). They were able to integrate their CAR on safe-harbor loci by electroporation of NK cells with CRISPR/Cas9 ribonucleoprotein targeting AAVS1 locus at day 7 of expansion, followed by AAV transduction. CAR sequence targeting CD33 was cloned into an AAV backbone, flanked by homology arms to allow CAR integration by homologous recombination, achieving more than 60% of CD33 CAR expression on electroporated NK cells. CD33 CAR-NK anti-tumor cytotoxicity was tested *in vitro* with tumor cell lines and primary samples obtained from AML patients, and showed enhanced cytotoxicity and cytokine secretion compared to non-modified NK cells.

### 4.6 Next-generation CAR-T cells with targeting module

Next-generation CAR-T cells have emerged as a solution to control the activation of CAR-T cells to reduce the chances of developing off-target toxicities. In this sense, authors have developed a platform of next-generation CAR-T cells with a chimeric receptor which recognizes a specific domain present on a targeting module (TM). This TM contains a binding domain, which can be directed against tumor-specific target antigens and mediates CAR-T antigen-specific activation. This strategy keeps the CAR inactivated until it reaches the TM, which only activates the CAR-T cells on the presence of antigen-expressing target cells (Loff et al., 2020), allowing a more specific activation towards target cells and reduced toxicity against healthy tissues.

Within this platform, some groups are trying to improve this technique to target leukemic cells in AML. Loff et al. engineered CAR-T cells to express a CAR to target a CD123-directed TM (CD123TM). These anti-CD123 strategy proved anti-leukemic capacity against leukemic cells *in vitro* and *in vivo*, with a cytotoxic capacity similar to a conventional CD123 CAR-T cell (Loff et al., 2020). They also proved their capacity to manufacture CD123TM and CAR-T cells under Good Manufacture Practice conditions with similar results than when generated in the laboratory, and it is currently being evaluated on a clinical trial including patients with R/R AML (NCT04230265) (Wermke et al., 2021; Ehninger et al., 2022).

### 4.7 Third-generation CAR-T cells

Third-generation CAR-T cells are designed to include two costimulatory domains in their structure. They are designed to increase signaling downstream CAR to achieve higher efficacies combining both signals, especially in diseases with low tumor burden (Tomasik et al., 2022). In this sense, third-generation CAR-T cells are being developed in AML preclinical context.

Liu et al. developed a third-generation CAR containing the scFv derived from CD33, hinge, and transmembrane domain from IgG1 and, 4-1BB and CD28 costimulatory domains fused to CD3ζ. Cytotoxic activity of 3G.CAR33-T cells versus 2G.CAR33-T cells, which contained just one costimulatory domain, was compared and increased killing capacity with 3G.CAR33-T cells and 2G.CD28.CAR33-T cells than with 2G.4-1BB.CAR33-T cells (Liu et al., 2022).

In the same way, Trad et al. developed a third-generation CAR targeting IL-1RAP (Trad et al., 2022). They demonstrated IL-1RAP overexpression on LSCs and blasts in AML. Afterwards, they were able to efficiently transduce T cells derived from AML patients with anti-IL-1RAP CAR, and IL-RAP CAR-T cells efficiently killed AML tumor lines and primary samples *in vitro* and *in vivo*, with no toxicity against healthy tissues or HSCs.

## 5 Positioning CAR-T cell therapy in AML: novel solutions for distinct obstacles

Despite improved frontline and salvage therapy options, a significant proportion of AML patients fail to achieve disease remission before HSCT, which hampers post-transplant outcomes. In this sense, CAR-T cell therapy has been proposed as an effective conditioning, as it would result in a less toxic and more specific regimen, and once remission is achieved, CAR-T cells can be eradicated with different strategies (García-Guerrero et al., 2020; Guercio et al., 2021).

Arai Y et al. have developed an anti-c-kit CAR-T cell, an antigen expressed in HSCs (Arai et al., 2018), with increased expression of CXCR4 to improve migration to the bone marrow. They have generated murine CAR-T cells with more than 90% of c-kit CAR expression, reaching more than 80% of cytotoxicity in co-culture experiments with c-kit-positive cell lines *in vitro*. They also performed *in vivo* experiments, reaching more than 60% of bone marrow infiltration (Arai et al., 2018).

Salman et al. (2019) developed third-generation CD4-specific CAR-T cells and CD4 CAR-NK cells, since CD4 is expressed in some AML subtypes, as a strategy to eradicate CD4^+^ residual AML before transplant. CD4 CAR-T and CD4 CAR-NK cells exert specific and dose-dependent anti-leukemic effect against AML cell lines and primary samples. In a xenogeneic mouse injected with the MOLM-13 cell line, CD4 CAR-T cells exhibit an 80%–100% of disease control, while CD4 NK-CAR achieved a 98% tumor reduction up to day 9 of treatment. CAR-T cell-based maintenance or pre-emptive therapies might be also used to decrease the risk of relapse after alloHSCT, which remains the major cause of transplant failure in AML. Here, the use of gene-editing technologies could help circumvent on-target toxicities through the deletion of target antigens known to be dispensable for healthy hematopoietic function in donor progenitor cells. Following engraftment of the gene-edited stem cells, CAR-T cell therapy could be then deployed sparing graft toxicity. This approach is being pursued in an industry-led phase I trial evaluating alloHSCT with a CRISPR-edited CD33-depleted allograft followed by maintenance with the anti-CD33 antibody-drug conjugate (ADC) gemtuzumab ozogamicin (NCT05662904) (Liu et al., 2022), with plans for subsequent trials using anti-CD33 CAR-T cell therapy. While CD33 is an interesting target for proof-of-concept purposes, as it appears to be functionally redundant in humans, additional targets may be suitable for this strategy. In fact, preclinical data suggest that deletion of both CD33 and CLL-1 does not result in impaired hematopoietic function (Xavier-Ferrucio et al., 2022). These findings open potential avenues for multi-target allograft edition followed by multi-specific (i.e., anti-CD33 and anti-CLL-1) CAR-T cell post-transplant therapy (Humbert et al., 2018; Kim et al., 2018; Xavier-Ferrucio et al., 2022).

## 6 Future directions and conclusions

Clinical response rates following CAR-T cell therapy in AML have been notably lower compared to those observed in B-cell malignancies. A key contribution to these disappointing results might be found in the autologous T-cell dysfunction, either as a result of prior lymphotoxic treatments or as a direct consequence of the immunosuppressive nature of the tumor microenvironment in AML (Lamble et al., 2020; Hao et al., 2021; Menter and Tzankov, 2022; Zarychta et al., 2023). Identifying target antigens exclusively expressed on AML blasts is also a significant challenge to avoid myelotoxicity resulting from target antigen expression on healthy cells. Despite these hurdles, several limitations must be addressed to enhance CAR-T cell therapy efficacy in AML. The development of new strategies, such as combining CAR-T cell therapy with different drugs to increase antigen density in AML cells, is imperative. Additionally, leveraging gene editing tools is a current area of research, enabling the modification of target antigen expression in HSCs before alloHSCT to mitigate off-target toxicities. Furthermore, the generation of new and optimized CAR-T cells, such as dual CAR-T cells or third-generation CAR-T cells with increased activation against tumor cells to prevent antigen scape, is essential. Generating allogeneic, off-the-shelf CAR-T cell products from healthy donors could offer more efficient antileukemic activity, although challenges remain, particularly in the R/R setting. Targeting AML during remission with low detectable burden (i.e., MRD) or undetectable disease could allow for enhanced CAR-T cell activity and improved tolerability.

In summary, optimizing CAR-T cell therapy in AML patients will likely require innovative strategies beyond those employed in B-cell malignancies, and possibly a distinct clinical positioning within the therapeutic landscape of the disease.

## References

[B1] Albinger N. Pfeifer R. Nitsche M. Mertlitz S. Campe J. Stein K. (2022). Primary CD33-targeting CAR-NK cells for the treatment of acute myeloid leukemia. Blood Cancer J. 12, 61. 10.1038/s41408-022-00660-2 35418180 PMC9007937

[B2] Alvarez-Fernández C. Escribà-Garcia L. Caballero A. C. Escudero-López E. Ujaldón-Miró C. Montserrat-Torres R. (2021). Memory stem T cells modified with a redesigned CD30-chimeric antigen receptor show an enhanced antitumor effect in Hodgkin lymphoma. Clin. Transl. Immunol. 10, e1268. 10.1002/CTI2.1268 PMC8082716 33968404

[B3] Arai Y. Choi U. Corsino C. I. Koontz S. M. Tajima M. Sweeney C. L. (2018). Myeloid conditioning with c-kit-Targeted CAR-T cells enables donor stem cell engraftment. Mol. Ther. 26, 1181–1197. 10.1016/J.YMTHE.2018.03.003 29622475 PMC5993968

[B4] Bachy E. Le Gouill S. Di Blasi R. Sesques P. Manson G. Cartron G. (2022). A real-world comparison of tisagenlecleucel and axicabtagene ciloleucel CAR T cells in relapsed or refractory diffuse large B cell lymphoma. Nat. Med. 28, 2145–2154. 10.1038/s41591-022-01969-y 36138152 PMC9556323

[B5] Baumeister S. H. Murad J. Werner L. Daley H. Trebeden-Negre H. Gicobi J. K. (2019). Phase I trial of autologous CAR T cells targeting NKG2D ligands in patients with AML/MDS and multiple myeloma. Cancer Immunol. Res. 7, 100–112. 10.1158/2326-6066.CIR-18-0307 30396908 PMC7814996

[B6] Budde L. E. Song J. Del Real M. Kim Y. Toribio C. Wood B. (2020). Abstract PR14: CD123CAR displays clinical activity in relapsed/refractory (r/r) acute myeloid leukemia (AML) and blastic plasmacytoid dendritic cell neoplasm (BPDCN): safety and efficacy results from a phase 1 study. Cancer Immunol. Res. 8, PR14. 10.1158/2326-6074.TUMIMM18-PR14

[B7] Calviño C. Ceballos C. Alfonso A. Jauregui P. Calleja-Cervantes M. E. San Martin-Uriz P. (2023). Optimization of universal allogeneic CAR-T cells combining CRISPR and transposon-based technologies for treatment of acute myeloid leukemia. Front. Immunol. 14, 1270843. 10.3389/FIMMU.2023.1270843 37795087 PMC10546312

[B8] Caruso S. De Angelis B. Del Bufalo F. Ciccone R. Donsante S. Volpe G. (2022). Safe and effective off-the-shelf immunotherapy based on CAR.CD123-NK cells for the treatment of acute myeloid leukaemia. J. Hematol. Oncol. 15, 163. 10.1186/S13045-022-01376-3 36335396 PMC9636687

[B9] Casirati G. Cosentino A. Mucci A. Salah Mahmoud M. Ugarte Zabala I. Zeng J. (2023). Epitope editing enables targeted immunotherapy of acute myeloid leukaemia. Nature 621, 404–414. 10.1038/s41586-023-06496-5 37648862 PMC10499609

[B10] Charrot S. Hallam S. (2019). CAR-T cells: future perspectives. Hemasphere 3, e188. 10.1097/HS9.0000000000000188 31723827 PMC6746028

[B11] Choi E. Chang J.-W. Krueger J. Lahr W. S. Pomeroy E. Walsh M. (2021). Engineering CD70-directed CAR-NK cells for the treatment of hematological and solid malignancies. Blood 138, 1691. 10.1182/BLOOD-2021-148649 34324630

[B12] Costa L. J. Lin Y. Cornell R. F. Martin T. Chhabra S. Usmani S. Z. (2022). Comparison of cilta-cel, an anti-BCMA CAR-T cell therapy, versus conventional treatment in patients with relapsed/refractory multiple myeloma. Clin. Lymphoma Myeloma Leuk. 22, 326–335. 10.1016/J.CLML.2021.10.013 34840088

[B13] Daver N. Alotaibi A. S. Bücklein V. Subklewe M. (2021). T-cell-based immunotherapy of acute myeloid leukemia: current concepts and future developments. Leukemia 35, 1843–1863. 10.1038/s41375-021-01253-x 33953290 PMC8257483

[B14] Dimitri A. Herbst F. Fraietta J. A. (2022). Engineering the next-generation of CAR T-cells with CRISPR-Cas9 gene editing. Mol. Cancer 21, 78. 10.1186/S12943-022-01559-Z 35303871 PMC8932053

[B15] DiNardo C. D. Cortes J. E. (2016). Mutations in AML: prognostic and therapeutic implications. Hematol. Am. Soc. Hematol. Educ. Program 2016, 348–355. 10.1182/asheducation-2016.1.348 PMC6142505 27913501

[B16] Döhner H. Wei A. H. Appelbaum F. R. Craddock C. DiNardo C. D. Dombret H. (2022). Diagnosis and management of AML in adults: 2022 recommendations from an international expert panel on behalf of the ELN. Blood 140, 1345–1377. 10.1182/blood.2022016867 35797463

[B17] Ediriwickrema A. Gentles A. J. Majeti R. (2023). Single-cell genomics in AML: extending the frontiers of AML research. Blood 141, 345–355. 10.1182/BLOOD.2021014670 35926108 PMC10082362

[B18] Ehninger A. Kramer M. Röllig C. Thiede C. Bornhäuser M. von Bonin M. (2014). Distribution and levels of cell surface expression of CD33 and CD123 in acute myeloid leukemia. Blood Cancer J. 4, e218. 10.1038/bcj.2014.39 24927407 PMC4080210

[B19] Ehninger G. Kraus S. Sala E. Metzelder S. K. Vucinic V. Fiedler W. (2022). Phase 1 dose escalation study of the rapidly switchable universal CAR-T therapy unicar-T-CD123 in relapsed/refractory AML. Blood 140, 2367–2368. 10.1182/BLOOD-2022-168877

[B20] el Khawanky N. Hughes A. Yu W. Myburgh R. Matschulla T. Taromi S. (2021). Demethylating therapy increases anti-CD123 CAR T cell cytotoxicity against acute myeloid leukemia. Nat. Commun. 12, 6436. 10.1038/S41467-021-26683-0 34750374 PMC8575966

[B21] Epperly R. Gottschalk S. Velasquez M. P. (2020). A bump in the road: how the hostile AML microenvironment affects CAR T cell therapy. Front. Oncol. 10, 262. 10.3389/FONC.2020.00262 32185132 PMC7058784

[B22] Fraietta J. A. Lacey S. F. Orlando E. J. Pruteanu-Malinici I. Gohil M. Lundh S. (2018). Determinants of response and resistance to CD19 chimeric antigen receptor (CAR) T cell therapy of chronic lymphocytic leukemia. Nat. Med. 24, 563–571. 10.1038/S41591-018-0010-1 29713085 PMC6117613

[B23] Freiwan A. Zoine J. T. Chase-Crawford J. Vaidya A. Schattgen S. A. Myers J. A. (2022). Engineering naturally occurring CD7^-^ T cells for the immunotherapy of hematological malignancies. Blood 140, 2684–2696. 10.1182/blood.2021015020 35914226 PMC9935551

[B24] García-Calderón C. B. Sierro-Martínez B. García-Guerrero E. Sanoja-Flores L. Muñoz-García R. Ruiz-Maldonado V. (2023). Monitoring of kinetics and exhaustion markers of circulating CAR-T cells as early predictive factors in patients with B-cell malignancies. Front. Immunol. 14, 1152498. 10.3389/fimmu.2023.1152498 37122702 PMC10140355

[B25] García-Guerrero E. Sierro-Martínez B. Pérez-Simón J. A. (2020). Overcoming chimeric antigen receptor (CAR) modified T-cell therapy limitations in multiple myeloma. Front. Immunol. 11, 1128. 10.3389/FIMMU.2020.01128 32582204 PMC7290012

[B26] Ghamari A. Pakzad P. Majd A. Ebrahimi M. Hamidieh A. A. (2021). Design and production an effective bispecific tandem chimeric antigen receptor on T cells against CD123 and folate receptor ß towards B-acute myeloid leukaemia blasts. Cell J. 23, 650–657. 10.22074/CELLJ.2021.7314 34939758 PMC8665988

[B27] Gomes-Silva D. Atilla E. Atilla P. A. Mo F. Tashiro H. Srinivasan M. (2019). CD7 CAR T cells for the therapy of acute myeloid leukemia. Mol. Ther. 27, 272–280. 10.1016/j.ymthe.2018.10.001 30391141 PMC6318703

[B28] Goswami M. Hourigan C. S. (2017). Novel antigen targets for immunotherapy of acute myeloid leukemia. Curr. Drug Targets 18, 296–303. 10.2174/1389450116666150223120005 25706110 PMC6321986

[B29] Guercio M. Manni S. Boffa I. Caruso S. Di Cecca S. Sinibaldi M. (2021). Inclusion of the inducible caspase 9 suicide gene in CAR construct increases safety of CAR.CD19 T cell therapy in B-cell malignancies. Front. Immunol. 12, 755639. 10.3389/FIMMU.2021.755639 34737753 PMC8560965

[B30] Halpern A. B. Rodríguez-Arbolí E. Othus M. Garcia K. A. Percival M. M. Cassaday R. D. (2023). Phase 1/2 study of sorafenib added to cladribine, high-dose cytarabine, G-CSF, and mitoxantrone in untreated AML. Blood Adv. 7, 4950–4961. 10.1182/bloodadvances.2023010392 37339483 PMC10463192

[B31] Hao F. Sholy C. Wang C. Cao M. Kang X. (2021). The role of T cell immunotherapy in acute myeloid leukemia. Cells 10, 3376. 10.3390/CELLS10123376 34943884 PMC8699747

[B32] Haubner S. Mansilla-Soto J. Nataraj S. Kogel F. Chang Q. de Stanchina E. (2023). Cooperative CAR targeting to selectively eliminate AML and minimize escape. Cancer Cell 41, 1871–1891. 10.1016/J.CCELL.2023.09.010 37802054 PMC11006543

[B33] He X. Feng Z. Ma J. Ling S. Cao Y. Gurung B. (2020). Bispecific and split CAR T cells targeting CD13 and TIM3 eradicate acute myeloid leukemia. Blood 135, 713–723. 10.1182/BLOOD.2019002779 31951650 PMC7059518

[B34] Hebbar N. Epperly R. Vaidya A. Thanekar U. Moore S. E. Umeda M. (2022). CAR T cells redirected to cell surface GRP78 display robust anti-acute myeloid leukemia activity and do not target hematopoietic progenitor cells. Nat. Commun. 13, 587. 10.1038/S41467-022-28243-6 35102167 PMC8803836

[B35] Hilpert J. Grosse-Hovest L. Grünebach F. Buechele C. Nuebling T. Raum T. (2012). Comprehensive analysis of NKG2D ligand expression and release in leukemia: implications for NKg2d-mediated NK cell responses. J. Immunol. 189, 1360–1371. 10.4049/JIMMUNOL.1200796 22730533

[B36] Hu Y. Zhou Y. Zhang M. Zhao H. Wei G. Ge W. (2022). Genetically modified CD7-targeting allogeneic CAR-T cell therapy with enhanced efficacy for relapsed/refractory CD7-positive hematological malignancies: a phase I clinical study. Cell Res. 32, 995–1007. 10.1038/S41422-022-00721-Y 36151216 PMC9652391

[B37] Huang R. Wen Q. Wang X. Yan H. Ma Y. Mai-Hong W. (2022). Off-the-Shelf CD33 CAR-NK cell therapy for relapse/refractory AML: first-in-human, phase I trial. Blood 140, 7450–7451. 10.1182/BLOOD-2022-170712

[B38] Humbert O. Laszlo G. S. Sichel S. Ironside C. Haworth K. G. Bates O. M. (2018). Engineering resistance to CD33-targeted immunotherapy in normal hematopoiesis by CRISPR/Cas9-deletion of CD33 exon 2. Leukemia 33, 762–808. 10.1038/s41375-018-0277-8 30291334

[B39] Jetani H. Garcia-Cadenas I. Nerreter T. Thomas S. Rydzek J. Meijide J. B. (2018). CAR T-cells targeting FLT3 have potent activity against FLT3−ITD+ AML and act synergistically with the FLT3-inhibitor crenolanib. Leukemia 32, 1168–1179. 10.1038/s41375-018-0009-0 29472720

[B40] Jin X. Zhang M. Sun R. Lyu H. Xiao X. Zhang X. (2022). First-in-human phase I study of CLL-1 CAR-T cells in adults with relapsed/refractory acute myeloid leukemia. J. Hematol. Oncol. 15, 88. 10.1186/S13045-022-01308-1 35799191 PMC9264641

[B41] Kantarjian H. Kadia T. DiNardo C. Daver N. Borthakur G. Jabbour E. (2021). Acute myeloid leukemia: current progress and future directions. Blood Cancer J. 11, 41. 10.1038/s41408-021-00425-3 33619261 PMC7900255

[B42] Kararoudi M. N. Likhite S. Elmas E. Yamamoto K. Schwartz M. Sorathia K. (2022). Optimization and validation of CAR transduction into human primary NK cells using CRISPR and AAV. Cell Rep. Methods 2, 100236. 10.1016/J.CRMETH.2022.100236 35784645 PMC9243630

[B43] Khawar M. B. Sun H. (2021). CAR-NK cells: from natural basis to design for kill. Front. Immunol. 12, 707542. 10.3389/FIMMU.2021.707542 34970253 PMC8712563

[B44] Kim M. Y. Yu K. R. Kenderian S. S. Ruella M. Chen S. Shin T. H. (2018). Genetic inactivation of CD33 in hematopoietic stem cells to enable CAR T cell immunotherapy for acute myeloid leukemia. Cell 173, 1439–1453. 10.1016/J.CELL.2018.05.013 29856956 PMC6003425

[B45] Knaus H. A. Berglund S. Hackl H. Blackford A. L. Zeidner J. F. Montiel-Esparza R. (2018). Signatures of CD8+ T cell dysfunction in AML patients and their reversibility with response to chemotherapy. JCI Insight 3, e120974. 10.1172/JCI.INSIGHT.120974 30385732 PMC6238744

[B46] Kuwana Y. Asakura Y. Utsunomiya N. Nakanishi M. Arata Y. Itoh S. (1987). Expression of chimeric receptor composed of immunoglobulin-derived V regions and T-cell receptor-derived C regions. Biochem. Biophys. Res. Commun. 149, 960–968. 10.1016/0006-291x(87)90502-x 3122749

[B47] Lagunas-Rangel F. A. Chávez-Valencia V. Gómez-Guijosa M. Á. Cortes-Penagos C. (2017). Acute myeloid leukemia—genetic alterations and their clinical prognosis. Int. J. Hematol. Oncol. Stem Cell Res. 11, 328–339. 29340131 PMC5767295

[B48] Lamble A. J. Kosaka Y. Laderas T. Maffit A. Kaempf A. Brady L. K. (2020). Reversible suppression of T cell function in the bone marrow microenvironment of acute myeloid leukemia. Proc. Natl. Acad. Sci. U. S. A. 117, 14331–14341. 10.1073/PNAS.1916206117 32513686 PMC7321988

[B49] Lee W. H. S. Ye Z. Cheung A. M. S. Goh Y. P. S. Oh H. L. J. Rajarethinam R. (2021). Effective killing of acute myeloid leukemia by TIM-3 targeted chimeric antigen receptor T cells. Mol. Cancer Ther. 20, 1702–1712. 10.1158/1535-7163.MCT-20-0155 34158344

[B50] Leick M. B. Silva H. Scarfò I. Larson R. Choi B. D. Bouffard A. A. (2022). Non-cleavable hinge enhances avidity and expansion of CAR-T cells for acute myeloid leukemia. Cancer Cell 40, 494–508.e5. 10.1016/J.CCELL.2022.04.001 35452603 PMC9107929

[B51] Li K. X. Wu H. Y. Pan W. Y. Guo M. Q. Qiu D. Z. He Y. J. (2022). A novel approach for relapsed/refractory FLT3mut+ acute myeloid leukaemia: synergistic effect of the combination of bispecific FLT3scFv/NKG2D-CAR T cells and gilteritinib. Mol. Cancer 21, 66. 10.1186/S12943-022-01541-9 35246156 PMC8896098

[B52] Lin G. Zhang Y. Yu L. Wu D. (2021). Cytotoxic effect of CLL-1 CAR-T cell immunotherapy with PD-1 silencing on relapsed/refractory acute myeloid leukemia. Mol. Med. Rep. 23, 208. 10.3892/MMR.2021.11847 33495835 PMC7830996

[B53] Liu F. Cao Y. Pinz K. Ma Y. Wada M. Chen K. (2018). First-in-Human CLL1-CD33 compound CAR T cell therapy induces complete remission in patients with refractory acute myeloid leukemia: update on phase 1 clinical trial. Blood 132, 901. 10.1182/BLOOD-2018-99-110579

[B54] Liu F. Zhang H. Sun L. Li Y. Zhang S. He G. (2020). First-in-human CLL1-CD33 compound CAR (cCAR) T cell therapy in relapsed and refractory acute myeloid leukemia. EHA Libr. 294969, S149.

[B55] Liu J. Zhou G. Zhang L. Zhao Q. (2019). Building potent chimeric antigen receptor T cells with CRISPR genome editing. Front. Immunol. 10, 456. 10.3389/FIMMU.2019.00456 30941126 PMC6433930

[B56] Liu Y. Wang S. Schubert M. L. Lauk A. Yao H. Blank M. F. (2022). CD33-directed immunotherapy with third-generation chimeric antigen receptor T cells and gemtuzumab ozogamicin in intact and CD33-edited acute myeloid leukemia and hematopoietic stem and progenitor cells. Int. J. Cancer 150, 1141–1155. 10.1002/IJC.33865 34766343

[B57] Loff S. Dietrich J. Meyer J. E. Riewaldt J. Spehr J. von Bonin M. (2020). Rapidly switchable universal CAR-T cells for treatment of cd123-positive leukemia. Mol. Ther. Oncolytics 17, 408–420. 10.1016/J.OMTO.2020.04.009 32462078 PMC7240059

[B58] Lynn R. C. Poussin M. Kalota A. Feng Y. Low P. S. Dimitrov D. S. (2015). Targeting of folate receptor β on acute myeloid leukemia blasts with chimeric antigen receptor-expressing T cells. Blood 125, 3466–3476. 10.1182/BLOOD-2014-11-612721 25887778 PMC4447861

[B59] Ma H. Padmanabhan I. S. Parmar S. Gong Y. (2019). Targeting CLL-1 for acute myeloid leukemia therapy. J. Hematol. Oncol. 12, 41. 10.1186/S13045-019-0726-5 31014360 PMC6480870

[B60] Ma Y.-J. Dai H.-P. Cui Q.-Y. Cui W. Zhu W.-J. Qu C.-J. (2022). Successful application of PD-1 knockdown CLL-1 CAR-T therapy in two AML patients with post-transplant relapse and failure of anti-CD38 CAR-T cell treatment. Am. J. Cancer Res. 12, 615–621. 35261791 PMC8899985

[B61] Mandal K. Wicaksono G. Yu C. Adams J. J. Hoopmann M. R. Temple W. C. (2023). Structural surfaceomics reveals an AML-specific conformation of integrin β2 as a CAR T cellular therapy target. Nat. Cancer 4, 1592–1609. 10.1038/S43018-023-00652-6 37904046 PMC10663162

[B62] Mardiana S. Gill S. (2020). CAR T cells for acute myeloid leukemia: state of the art and future directions. Front. Oncol. 10, 697. 10.3389/fonc.2020.00697 32435621 PMC7218049

[B63] Marvin-Peek J. Savani B. N. Olalekan O. O. Dholaria B. (2022). Challenges and advances in chimeric antigen receptor therapy for acute myeloid leukemia. Cancers (Basel) 14, 497. 10.3390/CANCERS14030497 35158765 PMC8833567

[B64] Maucher M. Srour M. Danhof S. Einsele H. Hudecek M. Yakoub-Agha I. (2021). Current limitations and perspectives of chimeric antigen receptor-T-cells in acute myeloid leukemia. Cancers (Basel) 13, 6157. 10.3390/cancers13246157 34944782 PMC8699597

[B65] Mehta P. H. Fiorenza S. Koldej R. M. Jaworowski A. Ritchie D. S. Quinn K. M. (2021). T cell fitness and autologous CAR T cell therapy in haematologic malignancy. Front. Immunol. 12, 780442. 10.3389/FIMMU.2021.780442 34899742 PMC8658247

[B66] Mehta R. S. Rezvani K. (2018). Chimeric antigen receptor expressing natural killer cells for the immunotherapy of cancer. Front. Immunol. 9, 283. 10.3389/fimmu.2018.00283 29497427 PMC5818392

[B67] Melenhorst J. J. Chen G. M. Wang M. Porter D. L. Chen C. Collins M. K. A. (2022). Decade-long leukaemia remissions with persistence of CD4+ CAR T cells. Nature 602, 503–509. 10.1038/s41586-021-04390-6 35110735 PMC9166916

[B68] Menter T. Tzankov A. (2022). Tumor microenvironment in acute myeloid leukemia: adjusting niches. Front. Immunol. 13, 811144. 10.3389/FIMMU.2022.811144 35273598 PMC8901718

[B69] Mitra A. Barua A. Huang L. Ganguly S. Feng Q. He B. (2023). From bench to bedside: the history and progress of CAR T cell therapy. Front. Immunol. 14, 1188049. 10.3389/fimmu.2023.1188049 37256141 PMC10225594

[B70] Molica M. Perrone S. Mazzone C. Niscola P. Cesini L. Abruzzese E. (2021). CD33 expression and gentuzumab ozogamicin in acute myeloid leukemia: two sides of the same coin. Cancers (Basel) 13, 3214. 10.3390/cancers13133214 34203180 PMC8268215

[B71] Molinos-Quintana A. Alonso-Saladrigues A. Herrero B. Caballero-Velázquez T. Galán-Gómez V. Panesso M. (2023). Impact of disease burden and late loss of B cell aplasia on the risk of relapse after CD19 chimeric antigen receptor T Cell (Tisagenlecleucel) infusion in pediatric and young adult patients with relapse/refractory acute lymphoblastic leukemia: role of B-cell monitoring. Front. Immunol. 14, 1280580. 10.3389/fimmu.2023.1280580 38292483 PMC10825008

[B72] Morgan M. A. Kloos A. Lenz D. Kattre N. Nowak J. Bentele M. (2021). Improved activity against acute myeloid leukemia with chimeric antigen receptor (CAR)-NK-92 cells designed to target CD123. Viruses 13, 1365. 10.3390/V13071365 34372571 PMC8310147

[B73] Naik S. Madden R. M. Lipsitt A. Lockey T. Bran J. Rubnitz J. E. (2022). Safety and anti-leukemic activity of cd123-CAR T cells in pediatric patients with AML: preliminary results from a phase 1 trial. Blood 140, 4584–4585. 10.1182/BLOOD-2022-170201

[B74] Network N. C. C. (2019). NCC clinical guidelines in oncology: acute myeloid leukemia. Natl. Compr. Cancer 17. 10.6004/jnccn.2019.0028

[B75] Ottaviano G. Georgiadis C. Gkazi S. A. Syed F. Zhan H. Etuk A. (2022). Phase 1 clinical trial of CRISPR-engineered CAR19 universal T cells for treatment of children with refractory B cell leukemia. Sci. Transl. Med. 14, eabq3010. 10.1126/SCITRANSLMED.ABQ3010 36288281

[B76] Pang Z. Wang Z. Li F. Feng C. Mu X. (2022). Current progress of CAR-NK therapy in cancer treatment. Cancers (Basel) 14, 4318. 10.3390/cancers14174318 36077853 PMC9454439

[B77] Pei K. Xu H. Wang P. Gan W. Hu Z. Su X. (2023). Anti‐CLL1‐based CAR T‐cells with 4‐1‐BB or CD28/CD27 stimulatory domains in treating childhood refractory/relapsed acute myeloid leukemia. Cancer Med. 12, 9655–9661. 10.1002/CAM4.5916 37031462 PMC10166968

[B78] Pelosi E. Castelli G. Testa U. (2023). CD123 a therapeutic target for acute myeloid leukemia and blastic plasmocytoid dendritic neoplasm. Int. J. Mol. Sci. 24, 2718. 10.3390/ijms24032718 36769040 PMC9917129

[B79] Pérez-Amill L. Armand-Ugon M. Peña S. Val Casals M. Santos C. Frigola G. (2022). CD84: a novel target for CAR T-cell therapy for acute myeloid leukemia. Blood 140, 7379–7381. 10.1182/BLOOD-2022-165339

[B80] Perna F. Berman S. H. Soni R. K. Mansilla-Soto J. Eyquem J. Hamieh M. (2017). Integrating proteomics and transcriptomics for systematic combinatorial chimeric antigen receptor therapy of AML. Cancer Cell 32, 506–519. 10.1016/J.CCELL.2017.09.004 29017060 PMC7025434

[B81] Png Y. T. Vinanica N. Kamiya T. Shimasaki N. Coustan-Smith E. Campana D. (2017). Blockade of CD7 expression in T cells for effective chimeric antigen receptor targeting of T-cell malignancies. Blood Adv. 1, 2348–2360. 10.1182/BLOODADVANCES.2017009928 29296885 PMC5729624

[B82] Qasim W. Zhan H. Samarasinghe S. Adams S. Amrolia P. Stafford S. (2017). Molecular remission of infant B-ALL after infusion of universal TALEN gene-edited CAR T cells. Sci. Transl. Med. 9, eaaj2013. 10.1126/SCITRANSLMED.AAJ2013 28123068

[B83] Rafiq S. Hackett C. S. Brentjens R. J. (2020). Engineering strategies to overcome the current roadblocks in CAR T cell therapy. Nat. Rev. Clin. Oncol. 17, 147–167. 10.1038/s41571-019-0297-y 31848460 PMC7223338

[B84] Ritchie D. S. Neeson P. J. Khot A. Peinert S. Tai T. Tainton K. (2013). Persistence and efficacy of second generation CAR T cell against the LeY antigen in acute myeloid leukemia. Mol. Ther. 21, 2122–2129. 10.1038/MT.2013.154 23831595 PMC3831035

[B85] Sallman D. A. DeAngelo D. J. Pemmaraju N. Dinner S. Gill S. Olin R. L. (2022a). Ameli-01: a phase I trial of UCART123v1.2, an anti-cd123 allogeneic CAR-T cell product, in adult patients with relapsed or refractory (R/R) CD123+ acute myeloid leukemia (AML). Blood 140, 2371–2373. 10.1182/BLOOD-2022-169928 36054916

[B86] Sallman D. A. Elmariah H. Sweet K. Mishra A. Cox C. A. Chakaith M. (2022b). Phase 1/1b safety study of prgn-3006 ultracar-T in patients with relapsed or refractory CD33-positive acute myeloid leukemia and higher risk myelodysplastic syndromes. Blood 140, 10313–10315. 10.1182/BLOOD-2022-169142

[B87] Sallman D. A. Kerre T. Havelange V. Poiré X. Lewalle P. Wang E. S. (2023). CYAD-01, an autologous NKG2D-based CAR T-cell therapy, in relapsed or refractory acute myeloid leukaemia and myelodysplastic syndromes or multiple myeloma (THINK): haematological cohorts of the dose escalation segment of a phase 1 trial. Lancet Haematol. 10, e191–e202. 10.1016/S2352-3026(22)00378-7 36764323

[B88] Salman H. Pinz K. G. Wada M. Shuai X. Yan L. E. Petrov J. C. (2019). Preclinical targeting of human acute myeloid leukemia using CD4-specific chimeric antigen receptor (CAR) T cells and NK cells. J. Cancer 10, 4408–4419. 10.7150/JCA.28952 31413761 PMC6691696

[B89] Scherer L. Tat C. Sauer T. Tashiro H. Naik S. Velasquez M. P. (2022). LigandCD70.CAR as a platform for dual-targeting CAR T cells for acute myeloid leukemia. Blood 140, 7396–7397. 10.1182/BLOOD-2022-170503

[B90] Schorr C. Perna F. (2022). Targets for chimeric antigen receptor T-cell therapy of acute myeloid leukemia. Front. Immunol. 13, 1085978. 10.3389/FIMMU.2022.1085978 36605213 PMC9809466

[B91] Shelikhova L. Rakhteenko A. Molostova O. Kurnikova E. Ukrainskaya V. Muzalevsky Y. (2022). Allogeneic donor-derived myeloid antigen directed CAR-T cells - for relapsed/refractory acute myeloid leukemia in children after allogeneic hematopoietic stem cell transplantation: report of three cases. Blood 140, 4600–4601. 10.1182/BLOOD-2022-168891

[B92] Soldierer M. Bister A. Haist C. Thivakaran A. Cengiz S. C. Sendker S. (2022). Genetic engineering and enrichment of human NK cells for CAR-enhanced immunotherapy of hematological malignancies. Front. Immunol. 13, 847008. 10.3389/FIMMU.2022.847008 35464442 PMC9022481

[B93] Sterner R. C. Sterner R. M. (2021). CAR-T cell therapy: current limitations and potential strategies. Blood Cancer J. 11, 69. 10.1038/s41408-021-00459-7 33824268 PMC8024391

[B94] Sugita M. Galetto R. Zong H. Ewing-Crystal N. Trujillo-Alonso V. Mencia-Trinchant N. (2022). Allogeneic TCRαβ deficient CAR T-cells targeting CD123 in acute myeloid leukemia. Nat. Commun. 13, 2227. 10.1038/S41467-022-29668-9 35484102 PMC9050731

[B95] Tambaro F. P. Singh H. Jones E. Rytting M. Mahadeo K. M. Thompson P. (2021). Autologous CD33-CAR-T cells for treatment of relapsed/refractory acute myelogenous leukemia. Leukemia 35, 3282–3286. 10.1038/S41375-021-01232-2 33833386 PMC8550958

[B96] Taussig D. C. Pearce D. J. Simpson C. Rohatiner A. Z. Lister T. A. Kelly G. (2005). Hematopoietic stem cells express multiple myeloid markers: implications for the origin and targeted therapy of acute myeloid leukemia. Blood 106, 4086–4092. 10.1182/BLOOD-2005-03-1072 16131573 PMC1895250

[B97] Testa U. Pelosi E. Frankel A. (2014). CD 123 is a membrane biomarker and a therapeutic target in hematologic malignancies. Biomark. Res. 2, 4. 10.1186/2050-7771-2-4 24513123 PMC3928610

[B98] Tomasik J. Jasiński M. Basak G. W. (2022). Next generations of CAR-T cells - new therapeutic opportunities in hematology? Front. Immunol. 13, 1034707. 10.3389/FIMMU.2022.1034707 36389658 PMC9650233

[B99] Trad R. Warda W. Alcazer V. Neto Da Rocha M. Berceanu A. Nicod C. (2022). Chimeric antigen receptor T-cells targeting IL-1RAP: a promising new cellular immunotherapy to treat acute myeloid leukemia. J. Immunother. Cancer 10, e004222. 10.1136/JITC-2021-004222 35803613 PMC9272123

[B100] Wang Q. S. Wang Y. Lv H. Y. Han Q. W. Fan H. Guo B. (2015). Treatment of CD33-directed chimeric antigen receptor-modified T cells in one patient with relapsed and refractory acute myeloid leukemia. Mol. Ther. 23, 184–191. 10.1038/MT.2014.164 25174587 PMC4426796

[B101] Wat J. Barmettler S. (2022). Hypogammaglobulinemia after chimeric antigen receptor (CAR) T-cell therapy: characteristics, management and future directions. J. Allergy Clin. Immunol. Pract. 10, 460–466. 10.1016/j.jaip.2021.10.037 34757064 PMC8837681

[B102] Wellhausen N. O’Connell R. P. Lesch S. Engel N. W. Rennels A. K. Gonzales D. (2023). Epitope base editing CD45 in hematopoietic cells enables universal blood cancer immune therapy. Sci. Transl. Med. 15, eadi1145. 10.1126/SCITRANSLMED.ADI1145 37651540 PMC10682510

[B103] Wermke M. Kraus S. Ehninger A. Bargou R. C. Goebeler M. E. Middeke J. M. (2021). Proof of concept for a rapidly switchable universal CAR-T platform with UniCAR-T-CD123 in relapsed/refractory AML. Blood 137, 3145–3148. 10.1182/BLOOD.2020009759 33624009 PMC8176767

[B104] Westwood J. A. Murray W. K. Trivett M. Haynes N. M. Solomon B. Mileshkin L. (2009). The Lewis-Y carbohydrate antigen is expressed by many human tumors and can serve as a target for genetically redirected T cells despite the presence of soluble antigen in serum. J. Immunother. 32, 292–301. 10.1097/CJI.0b013e31819b7c8e 19242371

[B105] Wu Z. Zhang H. Wu M. Peng G. He Y. Wan N. (2021). Targeting the NKG2D/NKG2D-L axis in acute myeloid leukemia. Biomed. Pharmacother. 137, 111299. 10.1016/j.biopha.2021.111299 33508619

[B106] Xavier-Ferrucio J. Luo C. Angelini G. Krishnamurthy S. Patel N. Pettiglio M. (2022). P1429: multiplex deletion of myeloid antigens CD33 and CLL-1 BY CRISPR/CAS9 in human hematopoietic stem cells highlights the potential of next-generation transplants for aml treatment. Hemasphere 6, 1312–1313. 10.1097/01.HS9.0000848572.60330.2F

[B107] Yao S. Jianlin C. Yarong L. Botao L. Qinghan W. Hongliang F. (2019). Donor-derived cd123-targeted CAR T cell serves as a RIC regimen for haploidentical transplantation in a patient with FUS-ERG+ AML. Front. Oncol. 9, 1358. 10.3389/FONC.2019.01358 31850234 PMC6901822

[B108] Yoshida T. Mihara K. Takei Y. Yanagihara K. Kubo T. Bhattacharyya J. (2016). All-trans retinoic acid enhances cytotoxic effect of T cells with an anti-CD38 chimeric antigen receptor in acute myeloid leukemia. Clin. Transl. Immunol. 5, e116. 10.1038/CTI.2016.73 PMC5192064 28090317

[B109] Zarychta J. Kowalczyk A. Krawczyk M. Lejman M. Zawitkowska J. (2023). CAR-T cells immunotherapies for the treatment of acute myeloid leukemia—recent advances. Cancers (Basel) 15, 2944. 10.3390/CANCERS15112944 37296906 PMC10252035

[B110] Zhang X. Yang J. Li J. Qiu L. Hongxing L. Xiong M. (2023). Naturally selected CD7-targeted chimeric antigen receptor (CAR)-T cell therapy for refractory/relapsed acute myeloid leukemia: phase I clinical trial. Blood 142, 218–219. 10.1182/blood-2023-179086

[B111] Zhu X. Li Q. Zhu X. (2022). Mechanisms of CAR T cell exhaustion and current counteraction strategies. Front. Cell Dev. Biol. 10, 1034257. 10.3389/FCELL.2022.1034257 36568989 PMC9773844

